# Demoralization: A concept analysis through a scoping review methodology

**DOI:** 10.1016/j.ijnsa.2024.100272

**Published:** 2024-11-28

**Authors:** Rongyu Hua, Patraporn Bhatarasakoon

**Affiliations:** Faculty of Nursing, Chiang Mai University, Chiang Mai, Thailand

**Keywords:** Demoralization, Concept analysis, Scoping review

## Abstract

**Background:**

Demoralization is a complex construct comprising of several clinical phenomena that has gained increasing interest in clinical practice and research; however, the concept needs to be sufficiently specified and clearly described. A concept analysis of demoralization is necessary to integrate previous research findings and establish the scientific foundation for future intervention research.

**Aim:**

To analyze the concept of demoralization in terms of its antecedents, attributes, consequences, and empirical referents in health.

**Methods:**

A concept analysis was performed using the Joanna Briggs Institute guidelines scoping review method and mapping information through the Walker and Avant concept analysis framework. Electronic databases, including PubMed, The Cochrane Library, Embase, PsycINFO, and Web of Science, were searched until September 15, 2024. The grey literature and other minor non-indexed publications were also reviewed.

**Results:**

A total of 106 articles were included in the review. Antecedents included medical illness, symptom burden, low social support and coping difficulties. Attributes were identified as follows: dysphoria, disheartenment, helplessness, hopelessness, loss of meaning and purpose, and sense of failure. Consequences included poor quality of life, depression, anxiety, suicidal ideation and desire to die. The concept of demoralization was illustrated through a model case, one exhibiting borderline criterion and another displaying challenge.

**Conclusion:**

This scoping review clarifies the clinical definition of demoralization, distinguishing it from common language usage and from other emotional symptoms frequently experienced by patients.

**Implications for Practice:**

The identified attributes of demoralization play vital roles in health assessments and should guide nurses in providing appropriate patient care early on. Interventions should address the concept's antecedents and consequences.

**What is already known**

• Demoralization is a complex construct comprised of several clinical phenomena.
• The concept of demoralization is variation in clinical practice and research.

Alt-text: Unlabelled box

**What this paper adds**

• Conceptual clarity about the term “demoralization” in health.
• Systematic assessment of the current evidence on confirming demoralization in health.
• Provide clearer boundaries that differentiate between the general term and the existing diagnostic tools.
• The identified attributes of demoralization play vital roles in health assessments and should guide healthcare providers in detecting the symptoms and providing appropriate patient care earlier.

Alt-text: Unlabelled box

## 1 Introduction

Demoralization is a complex construct comprised of several clinical phenomena that have sparked increased interest in clinical and research fields. Its prevalence was verified in clinical and non-clinical populations, contributing to generalizability across medical and psychiatric settings (Woźniewicz and Cosci, 2023). Research on demoralization primarily originates from cancer and palliative care literature, demoralization has been shown to affect 25 % to 35 % of patients (Robinson et al., 2015). Demoralization is of particular concern among patients as it is associated with several adverse consequences, such as decreased psychological well-being (Hsiu-Ling et al., 2021), reduced quality of life (Ghiggia et al., 2021) and a stronger predictor of suicidal ideation than depression (Vehling et al., 2017). Demoralization is defined in the Encarta World English Dictionary as the erosion or destruction of a person or group's courage, confidence, or hope. At a theoretical level, demoralization in medical patients appears to go beyond the conventional definition in dictionaries.

Multiple definitions of demoralization have been suggested since it was first introduced. Frank (1961) first introduced the term demoralization as a definite cluster of symptoms, a state akin to the “giving up-given up” complex, in which one primarily experiences persistent feelings of subjective incompetence or failure to meet one's own or others’ expectations, an inability to cope and problem solve. Schmale and Engel (1967) subsequently identified the so-called “giving up-given up” complex, a psychological state characterized by helplessness or hopelessness, the feeling of being at a loss and unable to cope. (Gruenberg, 1967) created the phrase “Social Breakdown Syndrome” to characterize the chronic demoralization in individuals suffering from long-term mental disorders. Frankl (1985) coined the term “suffering without meaning” to describe demoralization in death camps. Frank (1974) further described demoralization as characterized by feelings of impotence, isolation, and despair, which can contribute to diminished self-esteem and a sense of the meaninglessness of life. De Figueiredo and Frank (1982) viewed demoralization as a major public health issue and proposed the coexistence of distress and subjective incompetence. Clarke and Kissane considered demoralization as an abnormal response, characterized by loss of meaning and hopelessness, helplessness, and meaninglessness and was associated with suicidal ideation and the wish to die (Clarke and Kissane, 2002). These current definitions of demoralization, which have evolved from those previously described, are more specific and more readily differentiated from depression. The key focus in this particular conceptualization is on a loss of meaning and purpose (Robinson et al., 2016). Sansone argued that a certain level of demoralization may be a typical human reaction corresponding to the severity of mental or physical problems (Sansone and Sansone, 2010). There is frequently confusion between demoralization and depression, which raises lingering queries about how they are related. Current literature generally recognizes demoralization and depression as two distinct psychological conditions that can be experienced independently of one another but also in comorbidity with areas of symptom overlap. Some authors have described demoralization as a possible prodromal state of depression or suicidality (De Figueiredo and Gostoli, 2013) and suggested that demoralization may be examined as a risk factor in longitudinal studies.

As the concept of demoralization is still in flux, its boundaries are fuzzy, and there has been much debate about whether demoralization is a normal reaction to a state of overwhelming circumstances or a disorder. In addition, demoralization is a complex clinical phenomenon that necessitates a precise definition to differentiate it from its broader, everyday meaning. While often used colloquially to describe feelings of disappointment or discouragement, in the health context, demoralization encompasses specific components that set it apart from other emotional symptoms, such as depression or anxiety. Understanding this distinction is crucial to make precise diagnoses so treatments can effectively alleviate illness and disability (Angelino and Treisman, 2001). Unfortunately, this lack of accurate understanding hinders the early identification of and appropriate clinical intervention for demoralization. Patients with demoralization may be ignored or misdiagnosed, which could cause a series of negative consequences. In addition, the concept's vagueness may hinder healthcare providers in identifying its modifiable risk factors, which may increase the incidence of demoralization in patients. Given that, to reach a better consensus on the boundaries of the demoralization construct, how to reliably measure it, and in what way to integrate it within the current classification system and ultimately help improve quality of care by allowing clinicians to better tailor treatment towards restoring morale in patients, based on our searching, there was a concept analysis paper on the demoralization (Li et al., 2015), but it is not comprehensive and lacks a systematic procedure to identify and select studies in the review. Several systematic reviews on demoralization focus on the prevalence, risk factors (Wang et al., 2023a) and interventions (Dong et al., 2024) of demoralization.

There has yet to be a systematic assessment of these different viewpoints. The present study employs a scoping review approach to summarize, evaluate, and discuss the relevant definitions and research on demoralization. Scoping reviews employ clear, precise, and transparent research methods to minimize bias and provide a comprehensive overview of current knowledge (Peters et al., 2022). Therefore, this scoping review aims to clarify and analyze the concept of demoralization in patients following the Walker and Avant guidance.

## 2 Methods

### 2.1 Design

This scoping review was guided by the Joanna Briggs Institute guidelines scoping review methodology (Peters et al., 2024) combined with the process of concept analysis of Walker and Avant (2005) to map the demoralization concept. To do this involves eight stages: (1) selecting a concept; (2) determining the aim and purpose of the analysis; (3) identifying all uses of the concept that was discovered; (4) determining the defining attributes; (5) identifying a model case; (6) identifying additional cases; (7) identifying antecedents and consequences; and (8) defining empirical referents (Walker and Avant, 2005). This review addressed four main research questions: (1) What is demoralization in the health context? (2) What are the attributes of demoralization in the health context? (3) Which aspects precede and proceed the demoralization? (4) What measures or empirical referents are there for demoralization?

### 2.2 Select a concept

The concept of demoralization was selected due to its unclear, complicated nature. Over the past 40 years, a slowly growing body of research has attempted to define demoralization as a distinct clinical construct. However, there is still disagreement about the exact nature of demoralization. The absence of a clear definition could lead to confusion in both research and clinical practice. With an accurate concept, it is easier for researchers to develop a theoretical framework and tailored interventions to address demoralization. Given that a better understanding of demoralization would benefit patients, therefore, a concept analysis is used to examine the basic elements of demoralization to investigate its structure and function.

### 2.3 Determine the aim of the analysis

Concepts describe the phenomena of interest, and a better understanding and application of the concept can promote health and well-being in research. The strength of a concept depends on it being clearly defined (Walker and Avant, 2005). However, as a concept, demoralization is much talked about but still in flux, so the key step is to get a better consensus on the boundaries of the demoralization construct. Therefore, this concept analysis aims to develop an operational definition of demoralization within the context of healthcare studies.

### 2.4 Identify all uses of the concept discovered: literature review

We aimed to map the literature about demoralization, so we decided on a scoping review methodology guided by the JBI Scoping review (Peters et al., 2024). This systematic review focuses on central concepts and provides an overview of the current state of research.

#### 2.4.1 Data sources and search strategy

Relevant reports were identified by searching electronic databases, including The Cochrane Library, Embase, PubMed, PsycINFO, and Web of Science. Existing reviews were also checked for references, and the reference lists of eligible studies were searched by hand. The last search date was September 15, 2024, and the keyword used was (demoralization or demoralization syndrome or demorali*) across databases.

#### 2.4.2 Source of evidence selection

The articles used in this study met two criteria. They: (1) addressed at least one of the following: definitions, attributes, antecedents, consequences, and methods for demoralization measurement; and (2) were published in English.

Articles were excluded if they were:

1) Conference abstracts, protocol, letters, editorials, thesis, books, or case reports,
2) The concept was mentioned but not described/ defined fully: articles for which demoralization is only a minor focus,
3) The full text was unavailable.

Once the duplicate entries were deleted, two separate reviewers screened the titles and abstracts of all identified studies to eliminate ineligible articles. The eligibilities of the remaining publications were assessed by full-text examination by the same two independent reviewers. Any disagreements between the reviewers during the selection process were handled by consensus and discussion.

#### 2.4.3 Data extraction and analysis

Two reviewers did data extraction independently using the predesigned form. The following data were extracted from the selected studies: author, year of publication, country, title, design, population, sample size, measurement, definition, antecedents, attributes, and consequences. The direct citations related to the attributes, antecedents, and consequences of the demoralization were arranged in a literature matrix. A word cloud analysis was conducted before the data analysis to construct the concept based on the frequency used and the existing diagnostic criteria. The word cloud analysis (Voyant Tools was the software used for the text analysis) was used to examine the terms scholars most frequently used to describe the content related to demoralization in their studies. The word cloud analysis will be combined with the demoralization interview (DI) to define the number of attributes. The DI has good reliability and validity, with a threshold of 6 symptoms supporting a diagnosis of demoralization (Bobevski et al., 2022). A comparison of our attribute findings with the validated DI would strengthen the results considerably as the DI provides a categorical diagnosis of the condition. Last, the findings of the scoping review are presented in a table or figure.

### 2.5 Determine the defining attributes

Defining attributes are the clusters of characteristics that are most frequently associated with the concept and aid in distinguishing it from other similar concepts (Walker and Avant, 2005). First, we used word cloud analysis to identify characteristics associated with the concept that were frequently repeated. Next, based on the DI to define the number of attributes, the DI requires 6 out of 14 possible phenomena to be present to diagnose demoralization; therefore, our study included six attributes most frequently used from prior literatures.

### 2.6 Identify the model and additional cases

A model case is a case study that includes all the defining attributes of the concept (Walker and Avant, 2005). For additional cases, the borderline case and related cases will be used. A borderline case is an instance of the concept containing some but not all attributes. A related case is an example related to the concept but does not encompass all the defining attributes (Walker and Avant, 2005). We created these cases based on the extant literature reviewed and the research team's clinical and lived experiences. The cases are presented as narratives to capture all attributes, while the additional cases, i.e., borderline case and related case, will not convey all concept attributes (Walker and Avant, 2005). These three examples can help the researchers clarify the boundaries of the concept.

### 2.7 Identify antecedents and consequences

Antecedents refer to circumstances or events that must happen before the concept. They help to position the concept within its usual social context and determine the underlying assumptions of the concept (Walker and Avant, 2005). We categorized key points described in the literature as “predictors” or “influencing factors” of demoralization as antecedents. Consequences are the results of the concept and the effects or outcomes of the concept (Walker and Avant, 2005). Accordingly, we categorized key points described as “outcomes” of demoralization as consequences. Ultimately, word cloud analysis identifies the most common antecedents and consequences.

### 2.8 Define empirical referents

Empirical referents are classes or categories of actual phenomena that, by their existence or presence, demonstrate the occurrence of the concept itself, and the empirical referents relate directly to the defining attributes, not the entire concept itself (Walker and Avant, 2005). For this concept, the defining attributes were theoretical principles, and the empirical referents were tangible examples of operationalizing them.

## 3 Results

### 3.1 Characteristics of the articles included

**Fig. 1:**
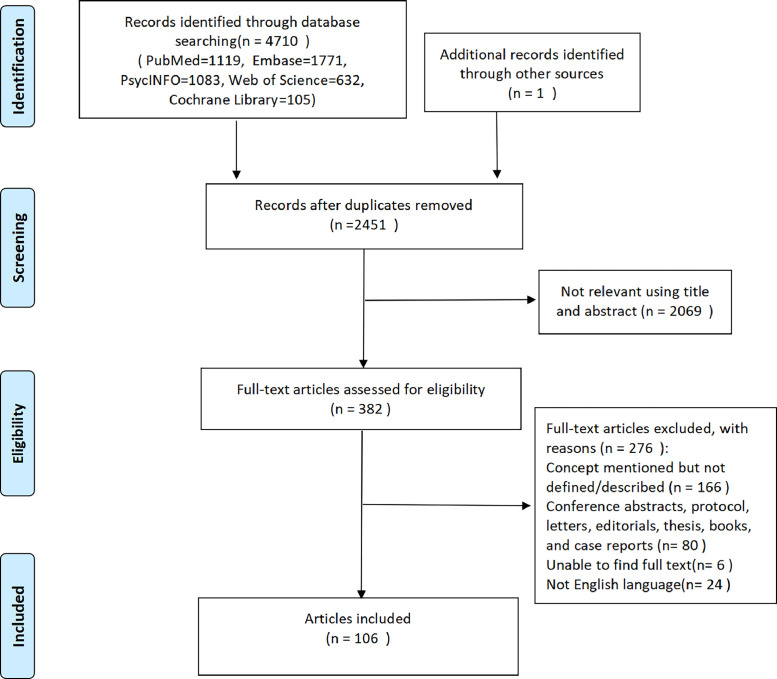
Flow chart of the study selection.

Database and grey literature searches retrieved 2451 documents post-deduplication. Fig. 1 provides a PRISMA illustration of the study inclusion/exclusion process and analysis approaches used.

As a result of the literature search, the 106 articles included in the analysis were published between 1974 and 2024. The studies were conducted in 13 countries, which were mainly distributed in China (n = 28, 26 %), United States (n = 26, 24 %), Australia and Italy had equal levels of representation (n = 15, 14 % each), Germany (n = 7, 7 %), Switzerland, Canada and Spain were equally represented (n = 3, 3 % respectively), Israel (n = 2, 2 %) and other countries (New Zealand, Iran, Greece, Brazil: n = 1,1 % respectively). The major design of included studies, consisting of a cross-sectional study (n = 68, 64 %), literature review (n = 15, 14 %), systematic review (n = 11, 10 %), qualitative study and case-control studies were equally represented (n =4, 4 % each), quasi-experimental study (n = 3, 3 %), randomized controlled trial (n = 1, 1 %). The major populations included patients with cancer (n = 48, 45 %), medical illness (n = 24, 22 %), general population (n = 10, 9 %), psychiatric patients (n = 5, 5 %), patients with Parkinson disease (n = 4, 4 %), refugee and migrant populations, clinical staff, transplant recipients including cardiac and kidney, family caregivers and acute coronary heart disease were equally represented (n = 2, 2 % each), and other populations (women in the perinatal period, American soldiers overseas, cardiac surgery patients, female Holocaust survivors, maintenance hemodialysis: n = 1, 1 % respectively). The sample size of included studies was from 7 to 10,641.

Half of the studies used the Demoralization Scale (DS, n = 48, 54 %), the next is Demoralization Scale-II (DS-II) (n = 12, 14 %) and Diagnostic Criteria for Psychosomatic Research (DCPR) (n = 11, 12 %). The remaining quantitative studies assessed demoralization through the following measurements: the Psychiatric Epidemiology Research Interview (PERI), Subjective Incompetence Scale (SIS), the Beck Depression Inventory (BDI), the Dysfunctional Attitudes Scale, Composite International Diagnostic Interview (CIDI), the Brief Cope Scale, Calgary Depression Scale for Schizophrenia (CDSS), Short Demoralization Scale (SDS), Demoralization Interview (DI), the Hopelessness Scale, Demoralization and Subjective Incompetence Scale (DSIS20). The detailed characteristics of the 106 included studies are shown in Table 1.

**Table 1:** Characteristics of included studies.

First author/Year, Country	Title	Design	Population (Sample size)	Measurement of demoralization	Definition	Antecedents	Attributes	Consequences
Frank, 1974, United States	Psychotherapy: The Restoration of Morale	Literature review	Medical illness (NA)	NA	Demoralization is experienced as a persistent inability to cope, together with associated feelings of helplessness, hopelessness, meaninglessness, subjective incompetence and diminished self-esteem	Medical illness	Feelings of helplessness, hopelessness, meaninglessness, subjective incompetence and diminished self-esteem, persistent inability to cope	NA
Dohrenwend et al., 1980, United States	Nonspecific psychological distress and other dimensions of psychopathology. Measures for use in the general population	Cross-sectional study	General population (200)	the Psychiatric Epidemiology Research Interview (PERI)	A constellation of feelings which include reduced self-esteem, helplessness and hopelessness, confused thinking, dread, anxiety, sadness, psychophysiological symptoms, and perceptions of poor physical health	NA	Diminished self-esteem, helplessness and hopelessness, confused thinking, dread, anxiety, sadness, psychophysiological symptoms, and perceptions of poor physical health	NA
De Figueiredo and Frank, 1982, United States	Subjective incompetence, the clinical hallmark of demoralization	Literature review	General population (NA)	NA	The coexistence of distress and subjective incompetence within a person	Stressful situation, inadequate social bonds, self-esteem	Distress, subjective incompetence	NA
Fenig and Levav, 1991, Israel	Demoralization and social supports among Holocaust survivors	Cross-sectional study	Female Holocaust survivors (145)	the Psychiatric Epidemiology Research Interview (PERI)	NA	low social supports, dose effect	NA	NA
Page and Cole, 1992, United States	Demoralization and living alone: outcome from an urban community study	Cross-sectional study	Urban adults (8634)	the Psychiatric Epidemiology Research Interview (PERI)	NA	Living alone	NA	NA
Stewart et al., 1993, United States	Demoralization predicts nonresponse to cognitive therapy in depressed outpatients	Quasi-experimental study	Depressed outpatients (39)	the Beck Depression Inventory (BDI), the Hopelessness Scale, the Dysfunctional Attitudes Scale	NA	NA	NA	Poor response of depression to cognitive therapy
Frank and Frank, 1996, United States	Demoralization and unexplained illness in two cohorts of American soldiers overseas	Literature review	American soldiers overseas (NA)	NA	Describe one reaction people exhibit when basic defensive maneuvers-symbolic mastery, escape, or attack-seem futile against a serious threat	Threatening situations	One reaction	inexplicable fatigue, rash, headache, arthralgias/ myalgias, difficulty concerntrating, shortness of breath, forgetfulness, irritability
Angelino and Treisman, 2001, United States	Major depression and demoralization in cancer patients: Diagnostic and treatment considerations	Literature review	Cancer patients (NA)	NA	The normal psychological reaction to life stresses and may be a disorder without implying a pathology of brain	Life stresses, having cancer	Normal psychological reaction, a disorder without implying a pathology of brain	Poor quality of life
Kissane et al., 2001, Australia	Demoralization syndrome–a relevant psychiatric diagnosis for palliative care	Literature review	Palliative care patients (NA)	NA	It is a form of psychological distress that presents due to a breakdown in coping and features hopelessness, helplessness, and a loss of meaning and purpose	A breakdown in coping	A form of psychological distress, hopelessness, helplessness, and a loss of meaning and purpose	Depression, desire for death
Clarke and Kissane, 2002, Australia	Demoralization: Its phenomenology and importance	Literature review	Medical illness (NA)	NA	Demoralization is experienced as existential despair, hopelessness, helplessness, and loss of meaning and purpose in life	Stressful situation	Existential despair, hopelessness, helplessness, loss of meaning and purpose	Poor outcomes in physical and psychiatric illness, suicidal ideation, the wish to die
Rickelman, 2002, United States	Demoralization as a precursor to serious depression	Literature review	General population (NA)	NA	Persons may become demoralized rather than depressed when they perceive that, at least temporarily, they cannot meet the demands placed on them by specific life stressors; they attribute their failure to internal or external deficits; and they manifest deficit coping in their cognitive, affective, and social responses.	Negative life events and coping difficulties	(Cognitive response) a sense of helplessness, perceived incompetence and lack of control, circumscribed (rather than generalized) pessimism, cognitive rigidity, decisional uncertainty, and avoidance of responsibility; (Affective response)anxiety, discouragement, frustration or anger, and somatic symptoms; (Social response) withdraw, avoid change, experience problematic interpersona lrelationships and some degree of social isolation, and feel a sense of alienation or lack of meaning in their lives	Suicide ideation, depression
Klssane, 2004, United States	The contribution of demoralization to end of life decision making	Literature review	Medical illness (NA)	NA	A mental state ranging from a normal response to perceived helplessness to a morbid form of existential distress	Chronically medically ill, the dying	A mental state, a normal response, perceived helplessness, existential distress	Acceptance of dying, desperation to die
Kissane et al., 2004, United States	The demoralization scale: A report of its development and preliminary validation	Cross-sectional study	Cancer patients (100)	Demoralization Scale	A distinct psychiatric disorder in which loss of meaning and hope can potentially spoil any sense of a worthwhile life and future. The demoralized patient expresses nonspecific dysphoria such as distress, irritability, guilt, or regret, and, out of a growing sense of disheartenment, loss of purpose and meaning can lead to helplessness, hopelessness, worthlessness, and a desire to die or to hasten death	NA	Loss of meaning, dysphoria, disheartenment, helplessness, sense of failure	Desire to die, hasten death.
Clarke et al., 2005, Australia	Demoralization, anhedonia and grief in patients with severe physical illness	Cross-sectional study	Cancer patients (271)	NA	Demoralization was characterized by feelings of being unable to cope, distress, apprehension, helplessness, hopelessness, personal failing and aloneness	Lack of family cohesiveness, trait anxiety, avoidance coping, past psychiatric history, confrontation coping, quality of significant relationships	Hopelessness, despairing, brooding, depressed, angry, pessimistic, discouraged, tearful, anxious, unable to cope and death wish	NA
Griffith and Gaby, 2005, United States	Brief psychotherapy at the bedside: Countering demoralization from medical illness	Qualitative study	Medical illness (7)	NA	Demoralization is the despair, helplessness, and sense of isolation that many patients experience when affected by illness and its treatments	Illness, treatments	Despair, helplessness, and sense of isolation	NA
Rafanelli et al., 2005, Italy	Stressful life events, depression and demoralization as risk factors for acute coronary heart disease	Case-Control Studies	Acute coronary heart disease (194)	Diagnostic Criteria for Psychosomatic Research (DCPR)	NA	Life events	NA	Acute myocardial infarction
Clarke et al., 2006, Australia	A qualitative examination of the experience of 'depression' in hospitalized medically ill patients	Qualitative study	Medical illness (49)	NA	Characterized by the experience of feeling unable to cope in a particular stressful situation, and feeling increasingly helpless, hopeless and despairing, with a concomitant sense of subjective incompetence, diminished personal esteem and value, and a perception of meaninglessness and pointlessness to life such that a person might want to ‘give up’	NA	Feelings of being unable to cope, helplessness, hopelessness and diminished personal esteem	NA
Briggs and Macleod, 2006, New Zealand	Demoralisation - A useful conceptualisation of non-specific psychological distress among refugees attending mental health services	Cross-sectional study	Adult refugee and migrant clients (64)	NA	Demoralisation is seen as a change in morale spanning a spectrum of mental attitudes from disheartenment (mild loss of confidence) through despondency (starting to give up) and despair (losing hope) to demoralisation (having given up)	Immediate family in New Zealand, somatic complaints	A spectrum of mental attitudes, disheartenment, despondency, despair	Suicidal behaviour, response to medication
Butterworth et al., 2006, Australia	Hopelessness, demoralization and suicidal behaviour: The backdrop to welfare reform in Australia	Cross-sectional study	Australian income support recipients (10641)	Composite International Diagnostic Interview (CIDI)	NA	Unemployment, lone mothers, disability payment recipients	NA	The capacity of individuals to engage in society
Boscaglia and Clarke, 2007, Australia	Sense of coherence as a protective factor for demoralisation in women with a recent diagnosis of gynaecological cancer	Cross-sectional study	Cancer patients (120)	Demoralization Scale	NA	Weak Sense of Coherence	NA	NA
Jacobsen et al., 2007, United States	Demoralization in medical practice	Literature review	Medical illness (NA)	NA	Demoralization in the medically ill refers to gamut of thoughts and negative emotions experienced by a patient when he or she unable to cope with life's adversities, treatment should target these unwelcome affects, behaviors, and cognitions	Pain	Gamut of thoughts and negative emotions	Psychological suffering, suicidal ideation
Marchesi and Maggini, 2007, Italy	Socio-demographic and clinical features associated with demoralization in medically ill in-patients	Cross-sectional study	Medical illness (296)	the Psychiatric Epidemiology Research Interview (PERI)	NA	Poor family support, severity of functional disability, number of threatening life events in the past year, female, depression, adjustment disorder	NA	NA
Wein, 2007, Australia	Is courage the counterpoint of demoralization?	Literature review	Palliative care patients (NA)	NA	NA	Courage	NA	NA
Cockram et al., 2009, United States	Diagnosis and measurement of subjective incompetence: the clinical hallmark of demoralization.	Cross-sectional study	Cancer patients (112)	the Brief Cope Scale, Subjective Incompetence Scale (SIS)	A demoralized person is discouraged, disheartened, bewildered and thrown into disorder or confusion	NA	Subjective incompetence	NA
Mullane et al., 2009, Israel	Validation of the Demoralization Scale in an Irish advanced cancer sample	Cross-sectional study	Cancer patients (100)	Demoralization Scale	It is a form of psychological distress that presents due to a breakdown in coping and features hopelessness, helplessness, and a loss of meaning and purpose	NA	Loss of meaning, dysphoria, disheartenment, sense of failure, hopelessness	Depression
Cockram et al., 2010, United States	Subjective incompetence as a clinical hallmark of demoralization in cancer patients without mental disorder	Cross-sectional study	Cancer patients (71)	Subjective Incompetence Scale (SIS)	The state of mind of a person deprived of spirit or courage, disheartened, bewildered, and thrown into disorder or confusion	Perceived stress	Subjective incompetence	NA
Silverstein et al., 2010, United States	Functional interpretations of sadness, stress and demoralization among an urban population of low-income mothers	Qualitative study	Low-income urban mothers (28)	NA	Demoralization related to feeling that problems beyond one's control	NA	Perceived loss of control over one's life circumstances, empowerment	NA
Wellen, 2010, United States	Differentiation between demoralization, grief, and anhedonic depression	Literature review	Medical illness (NA)	NA	Demoralization is a phenomenon in which a patient reaches a state of subjective incompetence, hopelessness, and helplessness that can lead to that devastating moment in which he or she feels the only recourse left is to give up	Physical or psychological illnesses	A state, subjective incompetence, hopelessness, and helplessness	Give up
Mehnert et al., 2011, Germany	Demoralization and Depression in Patients With Advanced Cancer: Validation of the German Version of the Demoralization Scale	Cross-sectional study	Medical illness (1102)	Demoralization Scale	States of existential distress and a self-perceived incapacity to deal effectively with a specific stressful situation	NA	Loss of meaning and purpose, disheartenment, dysphoria, and sense of failure	Anxiety, depression, distress
Vehling et al., 2011, Germany	Global meaning and meaning-related life attitudes: exploring their role in predicting depression, anxiety, and demoralization in cancer patients	Cross-sectional study	Cancer patients (270)	Demoralization Scale	NA	Global sense of meaning, goal seeking	NA	NA
Vehling et al., 2012, Germany	Is advanced cancer associated with demoralization and lower global meaning? The role of tumor stage and physical problems in explaining existential distress in cancer patients	Cross-sectional study	Cancer patients (270)	Demoralization Scale	NA	Number of physical problems, palliative treatment intention	NA	NA
Cavelti et al., 2012, Switzerland	Self-stigma and its relationship with insight, demoralization, and clinical outcome among people with schizophrenia spectrum disorders	Cross-sectional study	Outpatients with schizophrenia spectrum disorders (145)	Calgary Depression Scale for Schizophrenia (CDSS), Beck Depression Inventory-Revised (BDI-II)	NA	Self-stigma, insight	NA	More psychotic symptoms, lower functioning
Gabel, 2013, United States	Demoralization in Health Professional Practice: Development, Amelioration, and Implications for Continuing Education	Literature review	Physicians and other health professionals (NA)	NA	NA	Values related conflicts with larger social, organizational or bureaucratic systems. continuing education and continuing professional development programs	NA	Negative health care consequences
Vehling et al., 2013, Germany	Receiving Palliative Treatment Moderates the Effect of Age and Gender on Demoralization in Patients with Cancer	Cross-sectional study	Cancer patients (750)	Demoralization Scale	NA	Lack social support, younger,female, number of physical problems	NA	NA
Fang et al., 2014, China(Taiwan)	A correlational study of suicidal ideation with psychological distress, depression, and demoralization in patients with cancer	Cross-sectional study	Cancer patients (200)	Demoralization Scale	NA	Psychological distress	NA	Suicidal ideation
Vehling and Mehnert, 2014, Germany	Symptom burden, loss of dignity, and demoralization in patients with cancer: a mediation model	Cross-sectional study	Cancer patients (366)	Demoralization Scale	NA	Loss of dignity, number of physical problems	NA	NA
Bobevski et al., 2015, Australia	Postnatal demoralisation among women admitted to a hospital mother-baby unit: validation of a psychometric measure	Cross-sectional study	Women in the postnatal period (209)	Demoralization Scale	A psychological state characterized by experiences of distress and sadness, helplessness, subjective incompetence and hopelessness, in the context of a stressful situation.	Negative experiences of motherhood, functional impairment	Dysphoria/hopelessness, helplessness, loss of meaning and sense of failure	NA
Hocking and Sundram, 2015, Australia	Demoralisation syndrome does not explain the psychological profile of community-based asylum-seekers	Cross-sectional study	Asylum-seeker and refugee (131)	the Psychiatric Epidemiology Research Interview (PERI)	A construct that features hopelessness, meaninglessness, and existential distress	Major depressive disorder (MDD), posttraumatic stress disorder (PTSD), anxiety, low socioeconomic status, post-migration stress, isolation,Visa status	Hopelessness, meaninglessnes, existential distress	NA
Li et al., 2015, China (Taiwan)	Posttraumatic growth and demoralization after cancer: The effects of patients' meaning-making	Cross-sectional study	Cancer patients (200)	Demoralization Scale	NA	Less sense-making and benefit-finding or longer time-since-diagnosis, lower posttraumatic growth	NA	NA
Robinson et al., 2015, Australia	A systematic review of the demoralization syndrome in individuals with progressive disease and cancer: a decade of research	Systematic Review	Medical illness (4545)	Demoralization Scale	NA	Poorly controlled physical symptoms, inadequately treated depression, anxiety,reduced social functioning, unemployment, single status	NA	NA
Tang et al., 2015, China (Taiwan)	A Systematic Review and Meta-Analysis of Demoralization and Depression in Patients With Cancer	Systematic Review and Meta-Analysis	Cancer patients (NA)	NA	NA	NA	NA	High suicide risk, depression
Tecuta et al., 2015, United States	Demoralization: a systematic review on its clinical characterization	Systematic Review	Medical illness (NA)	NA	Demoralization appears to be a distinctive psychological state characterized by helplessness, hopelessness, giving up and subjective incompetence	Having cancer, clinical situation	A distinctive psychological state, helplessness, hopelessness, giving up and subjective incompetence	Stress and adverse health outcomes
Robinson et al., 2016, Australia	Refinement and revalidation of the demoralization scale: The DS-II internal validity	Cross-sectional study	Medical illness (211)	Demoralization Scale-II (DS-II)	A state of maladaptive coping, demoralization develops with symptoms of hopelessness and helplessness associated with loss of purpose and meaning in life.	NA	Meaning and purpose, Distress and coping ability	NA
Rudilla et al., 2016, Spain	Demoralization Scale in Spanish-Speaking Palliative Care Patients	Cross-sectional study	Medical illness (226)	Demoralization Scale	Patients as impotent, isolated, despairing, alienated, rejected, and with low self-esteem	NA	Loss of meaning, dysphoria, disheartenment, helplessness, sense of failure	NA
Fava et al., 2017, United States	Current psychosomatic practice	Literature review	General population (NA)	Diagnostic Criteria for Psychosomatic Research(DCPR)	A feeling state characterized by the perception of being unable to cope with some pressing problems and/or of lack of adequate support from others (helplessness). The individual maintains the capacity to react; The feeling state is prolonged and generalized (at least 1-month duration);A feeling state characterized by the consciousness of having failed to meet expectations associated with the conviction that there are no solutions for current problems and difficulties (hopelessness)	NA	Helplessness, hoplelessness, maintain the ability to react, The feeling state is prolonged and generalized	NA
Galiana et al., 2017, Spain	The short demoralization scale (SDS): a new tool to appraise demoralization in palliative care patients	Cross-sectional study	Palliative care patients (226)	Short Demoralisation Scale (SDS)	It is a form of psychological distress that presents due to a breakdown in coping and features hopelessness, helplessness, and a loss of meaning and purpose	NA	Loss of meaning, helplessness, disheartenment, dysphoria, and sense of failure	NA
Grassi et al., 2017, Italy	The factor structure and use of the Demoralization Scale (DS‐IT) in Italian cancer patients	Cross-sectional study	Cancer patients (194)	Demoralization Scale, Diagnostic Criteria for Psychosomatic Research(DCPR)	A combination of distress and subjective incompetence; loss of meaning and purpose in life; and cognitive attitudes of pessimism, hopelessness/helplessness, sense of being trapped, and personal failure; with associated features of social alienation or isolation and lack of support	NA	Disheartenment, sense of failure, dysphoria, loss of meaning/purpose	NA
Li et al., 2017, China(Taiwan)	Protective Factors of Demoralization among Cancer Patients in Taiwan: An Age-matched and Gender-matched Study	Cross-sectional study	Cancer patients (428)	Demoralization Scale	NA	Poor family support, lower monthly income	NA	NA
Vehling et al., 2017, Canada	The association of demoralization with mental disorders and suicidal ideation in patients with cancer	Cross-sectional study	Cancer patients (430)	Demoralization Scale	NA	NA	NA	Suicidal ideation
An et al., 2018, Canada	Demoralization and death anxiety in advanced cancer	Cross-sectional study	Cancer patients (307)	Demoralization Scale	NA	Symptom burden, social relatedness	NA	Death anxiety
Garzon et al., 2018, Germany	Perceived doctor-patient relationship and its association with demoralization in patients with advanced cancer	Cross-sectional study	Cancer patients (187)	Demoralization Scale	NA	Perceived doctor-patient relationship	NA	NA
Ko et al., 2018, China(Taiwan)	Demoralization Syndrome Among Elderly Patients with Cancer Disease	Cross-sectional study	Cancer patients (113)	Demoralization Scale	NA	Lower education, gynecological cancer	NA	Suicide ideation, depression, distress
Koo et al., 2018, United States	Demoralization in Parkinson disease	Cross-sectional study	Patients with Parkinson disease (180)	Diagnostic Criteria for Psychosomatic Research (DCPR),Demoralization Scale	NA	Having Parkinson disease, younger, single, motor dysfunction	NA	Depression
Liao et al., 2018, China(Taiwan)	Factors associated with demoralisation syndrome in patients before and after cardiac surgery	Cross-sectional study	Cardiac surgery patients (76)	Demoralization Scale	NA	Depression, retirement	NA	NA
Belar et al., 2019, Spain	Multicenter Study of the Psychometric Properties of the New Demoralization Scale (DS-II) in Spanish-Speaking Advanced Cancer Patients	Cross-sectional study	Cancer patients (150)	Demoralization Scale-II (DS-II)	Characterized by a sense of feeling trapped which limits coping ability, accompanied by a loss of the sense and meaning of life, of hope, of one's value in life, low moral, and reduced optimism	Greater religious practice,history of anxiety, history of depression	loss of purpose, dysphoria, discouragement, helplessness, the feeling of failure	NA
Bovero et al., 2019, Italy	Exploring demoralization in end-of-life cancer patients: Prevalence, latent dimensions, and associations with other psychosocial variables	Cross-sectional study	Cancer patients (235)	Demoralization Scale	Demoralization is an existential distress syndrome that consists of an incapacity of coping, helplessness, hopelessness, loss of meaning and purpose, and impaired self-esteem.	Spiritual well-being, dignity, low self-esteem, poor social connection, and a general sense of vulnerability, physical symptoms	Emotional distress and inability to cope, loss of purpose and meaning, worthlessness, sense of failure, dysphoria	Increase proximity to death and with impaired clinical condition
Cheng et al., 2019, China	Translation and psychometric properties for the Demoralization Scale in Chinese breast cancer patients	Cross-sectional study	Cancer patients (203)	Demoralization Scale	A maladaptive coping state with a sense of subjective incompetence	NA	Dysphoria & disheartenment, loss of meaning and purpose, sense of failure, helplessness	NA
Dischinger et al., 2019, United States	Loss of resources and demoralization in the chronically ill	Cross-sectional study	Medical illness (194)	Diagnostic Criteria for Psychosomatic Research (DCPR)	NA	Loss of resources, symptom severity	NA	NA
Vehling et al., 2019, Canada	Attachment security and existential distress among patients with advanced cancer	Cross-sectional study	Cancer patients (382)	Demoralization Scale	NA	Lower attachment security, physical symptom burden, attachment avoidance, attachment anxiety, male	NA	NA
Wu et al., 2019, China (Taiwan)	Quality of life, demoralization syndrome and health-related lifestyle in cardiac transplant recipients - a longitudinal study in Taiwan	Cross-sectional study	Cardiac transplant recipients (99)	Demoralization Scale	NA	NA	NA	Poor quality of life
Xu et al., 2019, China	Relationship of Suicidal Ideation With Demoralization, Depression, and Anxiety: A Study of Cancer Patients in Mainland China	Cross-sectional study	Cancer patients (303)	Demoralization Scale	NA	NA	NA	Suicidal ideation
Battaglia et al., 2020, Italy	The Use of Demoralization Scale in Italian Kidney Transplant Recipients	Cross-sectional study	Kidney transplant recipients (134)	Demoralization Scale;Diagnostic Criteria for Psychosomatic Research(DCPR)	Characterized by a combination of distress and subjective incompetence, the loss of meaning and purpose in life, the lack of perceived social support, a sense of being trapped and personal failure, a cognitive attitude of pessimism, and hopelessness/ helplessness	kidney transplant (KTRs), symptom burden, several life problems	Meaninglessness/ helplessness, disheartenment, dysphoria, sense of failure, distress, subjective incompetence, pessimism, trapped, lack of perceived social support	NA
Belvederi Murri et al., 2020, Italy	Assessing demoralization in medically ill patients: Factor structure of the Italian version of the demoralization scale and development of short versions with the item response theory framework	Cross-sectional study	Medical illness (473)	Diagnostic Criteria for Psychosomatic Research(DCPR), Demoralization Scale	Characterized by low morale, loss of meaning and purpose in life, feelings of hopelessness and helplessness, and sense of being trapped	NA	Disheartenment, Dysphoria, Sense of Failure, Loss of Meaning and Purpose	NA
Costanza et al., 2020, Switzerland	Demoralization and Its Relationship with Depression and Hopelessness in Suicidal Patients Attending an Emergency Department	Cross-sectional study	Emergency department patients (199)	Demoralization Scale	NA	NA	NA	Suicidal ideation
Elder et al., 2020, Australia	The demoralisation of nurses and medical doctors working in the emergency department: A qualitative descriptive study	Qualitative study	Emergency department clinicians (nurses and medical doctors) (12)	NA	NA	Workload and departmental activity, lack of support, inadequate resourcing, a mis-match between societal, organisational and staff expectations	NA	NA
Grassi et al., 2020, Italy	Exploring and assessing demoralization in patients with non-psychotic affective disorders	Cross-sectional study	Patients with ICD-10 diagnoses of mood, anxiety, stress-related disorders or other non-psychotic disorders (377)	Demoralization Scale;Diagnostic Criteria for Psychosomatic Research(DCPR)	Characterized by a combination of 1) distress and subjective incompetence; 2) loss of meaning and purpose in life; 3) cognitive attitudes of pessimism, and hopelessness/helplessness; 4) sense of being trapped and personal failure; with 5) associated features of lack of perceived social support	Patients with bipolar or unipolar major depression and personality disorders	Meaninglessness/ helplessness, disheartenment, dysphoria, sense of failure, distress, subjective incompetence, pessimism, trapped, lack of perceived social support	NA
Li et al., 2020, China	Current status of demoralization and its relationship with medical coping style, self-efficacy and perceived social support in Chinese breast cancer patients	Cross-sectional study	Cancer patients (375)	Demoralization Scale	NA	New rural cooperative medical insurance, without child, modified radical mastectomy, adaptive and effective coping style, resignation, low self-efficacy, low social support	NA	NA
Liu et al., 2020, China	Serial multiple mediation of demoralization and depression in the relationship between hopelessness and suicidal ideation	Cross-sectional study	Cancer patients (244)	Demoralization Scale-II (DS-II)	NA	Hopelessness	NA	Suicidal ideation, depression
Belvederi Murri et al., 2020, Italy	The relationship between demoralization and depressive symptoms among patients from the general hospital: network and exploratory graph analysis	Cross-sectional study	Medical illness (447)	Demoralization Scale	Demoralization is envisioned as a mental state characterized by a combination of distress (low morale, sadness, discouragement, and resentment) and poor coping (feelings of being trapped or stuck because of a sense of inability to plan and initiate concerted action toward one or more goals), which determine feelings of pointlessness, helplessness and hopelessness.	NA	Low mood and morale, loss of purpose, frustrated isolation, pointless, hopelessness, trapped, helplessness	NA
Tang et al., 2020, China	The differences and the relationship between demoralization and depression in Chinese cancer patients	Cross-sectional study	Cancer patients (296)	Demoralization Scale	NA	Resignation medical coping method, hopelessness, positive life orientation, low education level	NA	Poor quality of life
Koo et al., 2021, United States	Demoralization predicts suicidality in patients with cluster headache	Case-Control Studies	Cluster headache patients (235)	Diagnostic Criteria for Psychosomatic Research (DCPR),Demoralization Scale	NA	NA	NA	Suicidal ideation
Koranyi et al., 2021, Germany	Psychometric Evaluation of the German Version of the Demoralization Scale-II and the Association Between Demoralization, Sociodemographic, Disease- and Treatment-Related Factors in Patients With Cancer	Case-Control Studies	Cancer patients (620)	Demoralization Scale-II (DS-II)	Demoralization as a state of maladaptive coping characterized by a loss of purpose and meaning in life, low morale, low optimism, as well as helplessness and hopelessness	Aged between 18 and 49 years, divorced/separated, lung cancer patients, receiving no radiotherapy	Loss of meaning and purpose, low morale, low optimism, maladaptive coping, helplessness, hopelessness	NA
Peng et al., 2021, China (Taiwan)	The Mediation and Suppression Effect of Demoralization in Breast Cancer Patients After Primary Therapy: A Structural Equation Model	Case-Control Studies	Cancer patients (201)	Demoralization Scale	NA	Stress	NA	Sleep disturbance, psychological well-being
Sun et al., 2021, China (Taiwan)	The Effects of Logotherapy on Distress, Depression, and Demoralization in Breast Cancer and Gynecological Cancer Patients A Preliminary Study	Quasi-experimental study	Cancer patients (61)	Demoralization Scale	NA	Logotherapy	NA	NA
Zhu et al., 2021, Italy	Demoralization and Quality of Life of Patients with Parkinson Disease	Cross-sectional study	Patients with Parkinson disease (95)	Diagnostic Criteria for Psychosomatic Research (DCPR),Demoralization Scale	NA	Stigma, perceived difficulty with mobility	NA	Poor quality of life
Bobevski et al., 2022a, Australia	The Demoralization Interview: Reliability and validity of a new brief diagnostic measure among medically ill patients	Cross-sectional study	Medical illness (264)	Demoralization Scale-II (DS-II), Demoralization Interview (DI)	Demoralization is a mental state of low morale and poor coping, characterized by feeling trapped in a predicament or stressful event, where the further development of hopelessness, pointlessness, sense of failure and resultant isolation can lead to suicidal thinking	Illness	Not coping well, distress, low morale, failed to meet expectations, hopelessness, helplessness, trapped, pointlessness, loss of key roles, loss of confidence, not a worthwhile person, isolation, life no longer worth living, suicidal ideation or plans	Poor quality of life, physical disability, depressive and anxiety symptoms
Bobevski et al., 2022b, Australia	Demoralisation and its link with depression, psychological adjustment and suicidality among cancer patients: A network psychometrics approach	Cross-sectional study	Cancer patients (1529)	Demoralization Scale-II (DS-II)	It is defined by difficulty in adjusting to a stressor, wherein the person feels they are trapped in their predicament and experience feelings of helplessness, hopelessness, loss of confidence and loss of meaning in life	Having cancer	Trapped, helplessness, hopelessness, loss of confidence, loss of meaning	NA
Bovero et al., 2022, Italy	Demoralization in End-of-Life Cancer Patients' Family Caregivers: A Cross-Sectional Study	Cross-sectional study	Family Caregivers (142)	Demoralization Scale	NA	Spiritual and psychological suffering	NA	NA
Chang et al., 2022, China (Taiwan)	Frontal Lobe Functions, Demoralization, Depression and Craving as Prognostic Factors and Positive Outcomes of Patients with Heroin Use Disorder Receiving 6 Months of Methadone Maintenance Treatment	Quasi-experimental study	Patients with Heroin Use Disorder (39)	Demoralization Scale	NA	6-month course of MMT (Methadone maintenance therapy)	NA	NA
Chang et al., 2022, China (Taiwan)	Demoralization and Its Association with Quality of Life, Sleep Quality, Spiritual Interests, and Suicide Risk in Breast Cancer Inpatients: A Cross-Sectional Study	Cross-sectional study	Cancer patients (121)	Demoralization Scale	NA	Unmet bio-psycho-social-spiritual needs	NA	Poorer quality of life and sleep quality, higher risk of suicide, worst prognosis
Chien et al., 2022, China (Taiwan)	Exploring psychological resilience and demoralisation in prostate cancer survivors	Cross-sectional study	Cancer patients (122)	Demoralization Scale	NA	Lower cancer-specific self-efficacy; higher hormonal, bowel and urinary symptoms and bother, lower psychological resilience	NA	NA
Costanza et al., 2022, Switzerland	Demoralization in suicide: A systematic review	Systematic Review	Community dwellers, patients with somatic or psychiatric disorders (NA)	NA	NA	NA	NA	Suicidal ideation, suicidal behavior
De Figueiredo et al., 2022, United States	From Perceived Stress to Demoralization in Parkinson Disease: A Path Analysis	Cross-sectional study	Patients with Parkinson disease (95)	Demoralization Scale	NA	Perceived stress, subjective incompetence, depression, anxiety	NA	NA
Gan et al., 2022, Australia	Mental state of demoralisation across diverse clinical settings: A systematic review, meta-analysis and proposal for its use as a 'specifier' in mental illness	Systematic Review and Meta-Analysis	Medical illness (NA)	NA	NA	Physical health burden, lower education, lower education, female, younger, being single/non-partnered, having poor social support, less sense of their cancer diagnosis, found lower benefit from, experienced lower post-traumatic growth after diagnosis	NA	Poor quality of life, suicidal ideation
Hong et al., 2022, China	Understanding factors influencing demoralization among cancer patients based on the bio-psycho-social model: A systematic review	Systematic Review	Cancer patients (NA)	Demoralization Scale,Demoralization Scale-II (DS-II)	NA	More physical symptoms, hopelessness, hope, attachment security, sense of coherence, less social support	NA	Desire for death, dignity-related distress, depression, anxiety, psychological distress, suicidal ideation, poor quality of life
Lin et al., 2022, China	Demoralization profiles and their association with depression and quality of life in Chinese patients with cancer: a latent class analysis	Cross-sectional study	Cancer patients (874)	Demoralization Scale-II (DS-II)	NA	NA	NA	Depression and poor quality of life
Scandurra et al., 2022, Italy	A cross-sectional study on demoralization in prostate cancer patients: the role of masculine self-esteem, depression, and resilience	Cross-sectional study	Cancer patients (197)	Demoralization Scale-II (DS-II)	NA	Lower scores on masculine self-esteem, higher scores on depressive symptoms, resilience	NA	NA
Lu et al., 2022, China	Effects of perceived stigma on depressive symptoms and demoralization in maintenance hemodialysis patients: Self-warmth and self-coldness as mediators	Cross-sectional study	Maintenance hemodialysis (301)	Demoralization Scale	NA	Perceived stigma, self-warmth, self-coldness	NA	NA
Botto et al., 2023, Italy	Demoralization during the Italian quarantine due to 2019 coronavirus disease pandemic: prevalence and association with psychological well-being and coping strategies	Cross-sectional study	General population (1123)	Demoralization Scale	NA	Depressed mood, positive well-being, self-control, general health, vitality, problem-solving, and avoidance and religious avoidant coping strategies, female, older, without children and not working during quarantine, quarantine-related changes	NA	NA
Bovero et al., 2023, Italy	Relationship between demoralization and quality of life in end-of-life cancer patients	Cross-sectional study	Cancer patients (170)	Demoralization Scale-II (DS-II)	NA	NA	NA	Poor quality of life
Chan et al., 2023, China (Hong Kong)	Prevalence of and factors associated with demoralization among family caregivers of palliative care patients in Hong Kong	Cross-sectional study	Family Caregivers (94)	Demoralization Scale	NA	Depression, caregiving strain	NA	NA
De Figueiredo et al., 2023, United States	Differential impact of resilience on demoralization and depression in Parkinson disease	Cross-sectional study	Patients with Parkinson disease (95)	Demoralization Scale	NA	Low resilience	NA	NA
Elmasian et al., 2023, Greece	Psychometric Properties of the Greek Version of Demoralization Scale-II (DS-II) in Patients with Cancer	Cross-sectional study	Cancer patients (150)	Demoralization Scale-II (DS-II)	Feelings of despair, isolation, hopelessness, loss of meaning and existential distress are the core features of the definition of demoralization	NA	Despair, hopelessness, isolation, loss of meaning, existential distress	NA
Fava et al., 2023, United States	Distinguishing and treating demoralization syndrome in cancer: A review	Literature review	Cancer patients (NA)	NA	A prolonged and generalized (i.e., at least 1-month duration) feeling state characterized by the perception of being unable to cope with some pressing problems and/or of lacking adequate support from others (helplessness), and yet the individual maintains the capacity to react emotionally to stimuli.	Having cancer	Perception of being unable to cope with some pressing problems, helplessness	NA
Hao et al., 2023, China	Contribution of coping style to the association between illness uncertainty and demoralisation in patients with breast cancer: a cross-sectional mediation analysis	Cross-sectional study	Cancer patients (211)	Demoralization Scale	NA	Coping styles, illness uncertainty	NA	NA
Garcia et al., 2023, Brazil	Demoralization and spirituality in oncology: an integrative systematic review	Systematic Review	Cancer patients (NA)	NA	NA	Non-fulfillment of spiritual needs	NA	Proximity of death, growing frailty
Kang et al., 2023, China	Risk Factors of Demoralization Among Lung Cancer Patients in Mainland China	Cross-sectional study	Cancer patients (289)	Demoralization Scale	NA	Middle-aged and older, the new rural cooperative medical payment, multiple times of chemotherapy, lower family monthly income, avoidance dimension, surrender dimension, less social support, symptom, overall health and function	NA	NA
Kaneriya et al., 2023, United States	Distinguishing Distress in the Context of Aging: Demoralization vs. Depression	Literature review	Older adults (NA)	NA	Demoralization is characterized by a sense of hopelessness, helplessness and persistent inability to cope due to a loss of meaning and purpose in life	Social isolation, unemployment, physical symptom burden, inadequately treated mood and anxiety disorders, family support, higher income, secure interpersonal attachments	hopelessness, helplessness and persistent inability to cope, loss of meaning and purpose in life	Suicidal ideation
Wang et al., 2023b, China	Systematic Review of Interventions for Demoralization in Patients With Cancer	Systematic Review	Cancer patients (NA)	NA	NA	Psilocybin - assisted psychotherapy and psychological interventions	NA	NA
Wang et al., 2023, China	Prevalence, Associated Factors and Adverse Outcomes of Demoralization in Cancer Patients: A Decade of Systematic Review	Systematic Review	Cancer patients (NA)	NA	NA	Sociodemographic Factors: age, gender, education, marital status, religion, employment status, monthly income, number of children, having health insurance; Disease and Treatment-Related Factors: cancer stage, cancer type, treatment, time since diagnosis, radiotherapy, symptom burden, KPS score, fatigue, previous history of depression or anxiety; Other Factors: social support, family support, and favorable relationships to health care providers, Quality of life, Body image disturbance, Medical coping style, Preferred to discuss expected survival	NA	Suicidal ideation, dignity-related distress, sleep disturbance, psychological well-being, hope, work ability, mood disorders, and anxiety disorders
YuYu et al., 2023, China	The mediating effect of mindfulness on demoralization syndrome and quality of life of thyroid cancer patients: A correlational study	Cross-sectional study	Cancer patients (310)	Demoralization Scale	NA	Mindfulness	NA	Poor quality of life
Dong et al., 2024, China	A Systematic Review of Interventions for Demoralization in Patients with Chronic Diseases	Systematic Review	Medical illness (NA)	NA	NA	Evidence-based meaning-centered psychotherapy, dignity therapy, psilocybin-assisted psychotherapy	NA	NA
Gostoli et al., 2024, Italy	Demoralization in acute coronary syndrome: Treatment and predictive factors associated with its persistence	Randomized controlled trial	Acute Coronary Syndromes (ACS) patients (91)	Diagnostic Criteria for Psychosomatic Research (DCPR)	NA	Cognitive-Behavioral and Well-Being therapies (CBT/WBT), somatization, history of depression	NA	NA
Lin and Her, 2024, China (Taiwan)	Demoralization in cancer survivors: an updated systematic review and meta-analysis for quantitative studies	Systematic Review and Meta-Analysis	Cancer patients (2902)	NA	NA	Females, elder patients, breast cancer survivors	NA	suicide ideation, suicide risk, anxiety and quality of life
Taghilou et al., 2024, Iran	Determining psychometric properties of the Persian version of demoralization scale-II in patients with cancer	Cross-sectional study	Cancer patients (170)	Demoralization Scale-II (DS-II)	Demoralization is a maladaptive coping characterized by a loss of purpose and meaning in life, low morale, low optimism, helplessness, and hopelessness	NA	Maladaptive coping, loss of meaning and purpose, low morale, low optimism, helpnessless, hopelessness	NA
Belvederi Murri et al., 2024, Italy	A tale of two constructs: combined assessment of demoralization and subjective incompetence	Cross-sectional study	General population (414)	Demoralization Scale, Subjective Incompetence Scale (SIS), Demoralization and Subjective Incompetence Scale (DSIS20)	Demoralization includes up to seven dimensions, namely dysphoria, disheartenment, loss of confidence, subjective incompetence, loss of meaning, hopelessness/ helplessness, social disconnectedness, desire to die	NA	Disheartenment, sense of failure, helplessness, irritability, loss of purpose, subjective incompetence	NA

NA means that the included articles did not provide the content.

### 3.2 Concept analysis

#### 3.2.1 Determine the defining attributes

**Fig. 2:**
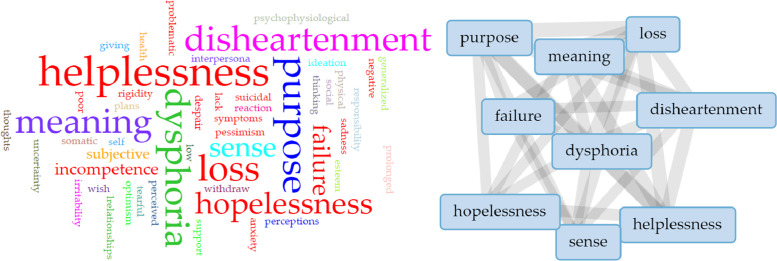
Word cloud analysis for attributes related to descriptions of included studies.

The word cloud analysis from the literature (Fig.2) showed that six defining attributes were most frequently associated with demoralization, differentiating it from similar concepts, such as depression. These attributes were dysphoria, disheartenment, helplessness, hopelessness, loss of meaning and purpose, and sense of failure.

**The details of each attribute are as follows.**

**1. Dysphoria**

Dysphoria is a nonspecific emotion of distress, regret, unease, restlessness, sadness, discomfort and unable to cope with stress, which can increase the risk of suicide (Kissane et al., 2004). It may occur in various clinical settings, often associated with different mental health conditions.

**2. Disheartenment**

The primary characteristic of disheartenment is experiencing discouragement, feeling trapped, isolated, or alone, and increasingly distressed about the situation (Kissane et al., 2004). Kissane et al. claim that disheartenment differs from the usual indicators observed by psychiatrists in diagnosing depression(Kissane et al., 2004).

**3. Helplessness**

Helplessness is when a person believes they are unable to change their stressful situation. Feelings of powerlessness and constraints from the illness are components of the helplessness felt by patients with medical or mental health conditions (Clarke et al., 2005).

**4. Hopelessness**

Hopelessness is little or no belief in a positive future. The person believes they are accountable for the predicament and lacks recourse or support to resolve it. Frank's work emphasized the crucial role of hope in therapy, underlining the significance of how individuals view the future and how a pessimistic outlook can greatly impact the human mind. Frank (1968). Clarke and Kissane pointed out that hopelessness is the hallmark of demoralization (Clarke and Kissane, 2002) and is associated with poor outcomes in physical and psychiatric illness and, importantly, with suicidal ideation and the desire to die (Robinson et al., 2016).

**5. Loss of meaning and purpose**

The meaning of life is a philosophical inquiry into the roles, achievements, and sources of satisfaction in an individual's existence (Lethborg et al., 2006). Beliefs encompass assumptive world, attitudinal values, global meaning, and generalized hope. These beliefs offer a sense of security when our ability to control things is jeopardized and questioned by circumstances like severe illness. Without the assumptive world, there is no reference point for the individual to draw between what is perceived as meaningful versus meaningless (Clarke and Kissane, 2002). Frank realized that reformulating a situation's meaning can change existing beliefs and objectives, potentially boosting positive emotions during challenging circumstances (Frank, 1974). The loss of meaning and purpose can be described as a loss of role, point to life, and sense of worth of life (Bobevski et al., 2022a, Kissane et al., 2004a).

**6. Sense of failure**

Sense of failure that can lead to a growing urge to give up, withdraw, or consider ending one's life because a worthwhile future is inconceivable (Kissane et al., 2001). A sense of accomplishment and success in life which, when reversed, signifies a “sense of failure” (Kissane et al., 2004a).

#### 3.2.2 2.2.2. Develop a model case

A model case contains all the necessary attributes including dysphoria, disheartenment, helplessness, hopelessness, loss of meaning and purpose, and sense of failure of demoralization.

A 34-year-old divorced and resigned woman, ‘‘Ms. A’’ was afflicted with breast cancer. She had a bilateral mastectomy. After the operation, she had chemotherapy and lost her hair despite being provided with a wig to keep up appearances. She was often too bothered to wear this and easily got upset and moody to ordinary event. Highly distressed by her disfigurement (dysphoria), she withdrew socially, only finding enjoyment when spending time with her family. She felt so trapped about why she got breast cancer (disheartenment). She felt no hope to get cured from the disease (hopelessness), and felt there is no one could help her get out of this situation (helplessness). She declared, “What is the point of going on? Everything I have done is over (loss of meaning and purpose); I am a loser. Life is no longer worth living; I just waste my time here and only wait to die (sense of failure).’’

#### 3.2.3 Develop additional cases

##### 3.2.3.1 Borderline case

A borderline case is an instance of demoralization that contains some but not all its attributes. In this example, the borderline case includes three attributes: hopelessness, disheartenment, and helplessness.

Ms. B, a married, thirty-five-year-old suffering from breast cancer and related lymphedema, caused her oncologist concern when she refused the recommended care (hopelessness). She appeared very discouraged as she spoke of her uncertain future, her frustration at a considerably restricted exercise tolerance, and the boredom that set in at home (disheartenment). She further told her oncologist, “There is no miracle in this world? My breast is getting worse; no one truly can help me (helplessness).”

##### 3.2.3.2 Related case: Anhedonic depression

In our study, the related case is anhedonic depression, which is related to demoralization but does not encompass all the defining attributes. Anhedonia, a central feature of depression, represents a multifaceted symptomatology that includes deficits in the capacity to experience pleasure, reduced approach-oriented motivated behaviour, and impaired learning related to environmental rewards (Treadway and Zald, 2011). It aids in promptly distinguishing anhedonic depression from demoralization (Clarke et al., 2005). A patient with anhedonic depression often feels an internal inability to enjoy life. In contrast, a demoralized patient is usually able to identify an external stressor that hinders their enjoyment of specific pleasurable activities (Mangelli et al., 2005).

Ms C is a 53-year-old married woman with a history of recurrent ovarian cancer and has undergone several treatments, including surgery and chemotherapy. During the past two years, she has had more than ten times hospitalizations and struggled to cope with her poor prognosis. During the assessment, the patient mentioned that she would prefer to be dead if the disease kept recurring like this while her husband was seated beside her, with little change in her tone or expression (emotional numbness). She exhibited a negative outlook and had begun to refuse essential nursing care and physical therapy during her hospitalization (diminished motivation). She often felt physically and emotionally exhausted, which has contributed to her decreased motivation. She experienced insomnia, frequently waking during the night and feeling unrested upon waking. In addition, she attributed her anorexia and substantial weight loss to her depressive state (physical symptoms). Her husband noted that even mentioning her favorite travel could not brighten her mood (anhedonia). She told him she could not join the trip because she would not be the same person. Her relationships with her husband have become strained. Her husband expressed concern about her withdrawal and inability to engage in family activities (impact on daily life). She stated, “I feel like a burden to my family,” she struggled with negative thoughts about her illness and future (cognitive deficits), and often thought, “Why should I try if I don't know how much time I have left? (This is the ability to identify the external stressor, i.e. her illness, that influenced and made her hold herself to live a full life).”

#### 3.2.4 Identify antecedents and consequences

##### 3.2.4.1 Antecedents

**Fig. 3:**
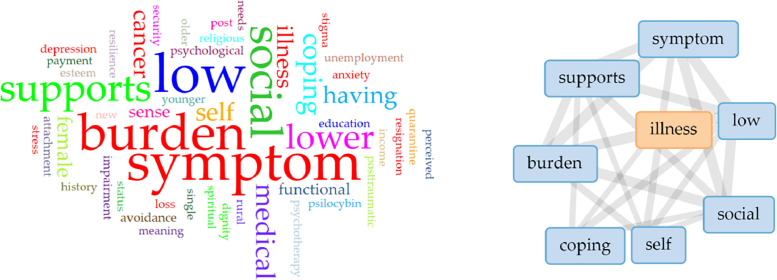
Word cloud analysis for antecedents-related descriptions of included studies.

The word cloud analysis from the literature matrix (Fig.3) identified medical illness, symptom burden, low social support and coping difficulties as the most frequently used to describe the antecedents of demoralization.

##### 3.2.4.2 Consequences

**Fig. 4:**
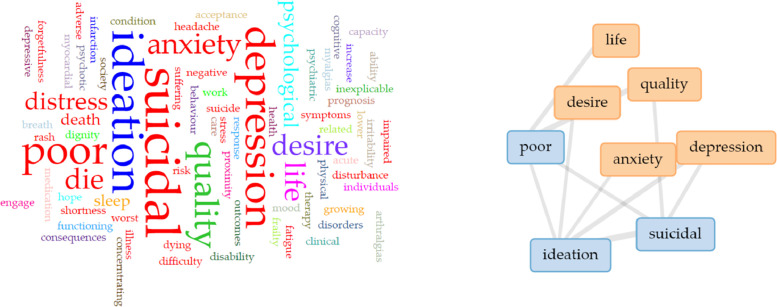
Word cloud analysis for consequence-related descriptions of included studies.

According to the word cloud analysis (Fig.4.), poor quality of life, depression, anxiety, suicidal ideation and desire to die have been commonly used in demoralization research to define the consequences of demoralization. Demoralization was found to uniquely contribute to an increased risk for suicidal ideation beyond the impact of a comorbid psychiatric disorder. Several literature reviews revealed correlations between demoralization and suicide risk and quality of life (Lin and Her, 2024), and some results emphasize the possibility of identifying demoralization as an independent risk factor for suicide (Costanza et al., 2022). Kissane also found in both the physically and mentally ill, as suffering develops, a severe form of demoralization leads to a “desire to die” (Kissane, 2001). The demoralization of patients with cancer was highly correlated with depression. If healthcare provider**s** can perceive patient's demoralization issues earlier, they can more effectively prevent patients’ depression from occurring, which benefits suicide prevention.

#### 3.2.5 Define empirical referents

Identifying empirical evaluation indicators allows observers to identify or measure the defining attributions (Walker and Avant, 2005). There are several assessment instruments designed to measure demoralization. The PERI (Psychiatric epidemiology research interview PERI) consists of 25 subscales, with eight scales designed to capture nonspecific distress (Dohrenwend et al., 1980). Given that the latest conceptualization of demoralization is focused on a loss of meaning and hope, the PERI has yet to be used to measure demoralization in recent research. The Diagnostic Criteria for Psychosomatic Research (DCPR) is a categorically-oriented, semi-structured interview that stems from this perspective and defines demoralization as the presence of the following symptoms for at least 1 month and occurring before a change in physical illness: feelings of helplessness, hopelessness, desire to give up, sense of inability to cope, perceived sense of not meeting the expectations of self and others (Porcelli and Rafanelli, 2010).

The Demoralization Scale (DS), designed by Kissane and colleagues (Kissane et al., 2004a), is a psychometric tool with 24 items and five factors: loss of meaning and purpose, dysphoria, disheartenment, helplessness, and sense of failure. A revalidation of the DS (DS-II) was proposed, which has 16 items and two components: meaning and purpose, distress, and coping ability. The meaning and purpose subscale combines items from the loss of meaning and purpose and helplessness subscales in the original DS into a single factor. DCPR is recommended to conduct macro-analysis. DS can be used as a complementary tool for micro-analysis, allowing for the specification of features such as loss of meaning and purpose, disheartenment, and sense of failure (Woźniewicz and Cosci, 2023). Recently, Belvederi Murri et al. (2024) combined selected items of the DS and the subjective incompetence scale (SIS) to develop the Demoralization and Subjective Incompetence Scale (DSIS20), a shorter measurement that includes subjective incompetence and other clinical components (disheartenment, sense of failure, helplessness, irritability, loss of purpose) of demoralization. However, further research may need to determine whether DSIS20 can assist in detecting demoralization early.

Demoralization Interview (DI), designed by Bobevski et al. (2022)a, which has 14 items and 14 symptoms: not coping well, distress, low morale, failure to meet expectations, hopelessness, helplessness, trapped, pointlessness, loss of key roles, loss of confidence, not a worthwhile person, isolation, life no longer worth living, suicidal ideation or plans. A diagnosis of demoralization will be made with a positive response to 6 of the 14 symptoms in this structured interview. The DI has good reliability and validity and could be used as a stand-alone diagnosis or as a specifier for adjustment disorder or depression (Bobevski et al., 2022a).

### 3.3 Definition

Demoralization, a state of mind, is attributed to the perception of dysphoria, disheartenment, helplessness, hopelessness, loss of meaning and purpose, and a sense of failure that arises from these factors: medical illness, symptom burden, low social support and coping difficulties. The demoralization could lead to poor quality of life, depression, anxiety, suicidal ideation and desire to die (Table 2).

**Table 2:** shows the attributes, antecedents, and consequences of demoralization.

Antecedents	Attributes	Consequences
medical illness	dysphoria	poor quality of life
symptom burden	disheartenment	depression
low social support	helplessness	anxiety
coping difficulties	hopelessness	desire to die
	loss of meaning and purpose	suicidal ideation
	sense of failure	

## 4 Discussion

In this review, we have identified the defining attributes, antecedents, consequences, and empirical referents of demoralization through a systematic search of the academic literature and a targeted search of the grey literature to present the current state of the science. The review revealed several central attributes of demoralization, including dysphoria, disheartenment, helplessness, hopelessness, loss of meaning and purpose, and sense of failure. Demoralization is not officially included in the current psychiatric classification system of the Diagnostic and Statistical Manual of Mental Disorders, fifth edition (DSM-5) (Kaneriya et al., 2023). In contrast, the ICD-11 (International Classification of Diseases for Mortality and Morbidity Statistics, 11th Revision, v2024-01) has included the diagnosis of demoralization (Code MB22.2), characterized as loss of confidence in one's ability to cope, with associated feelings of helplessness, hopelessness, and discouragement (WHO, 2023). Bobevski and associates (Bobevski et al., 2022b) also developed a 14-item DI to assess for and diagnose demoralization, which 14 possible symptoms, including not coping well, distress, low morale, failed to meet expectations, hopelessness, helplessness, trapped, pointlessness, loss of key roles, loss of confidence, not a worthwhile person, isolation, life no longer worth living, suicidal ideation or plans. These elements are crucial and require 6 out of 14 possible symptoms to be present to diagnose demoralization. The DI symptoms were broader than WHO definition. In our study, six attributes of demoralization were identified by the DI cut-off points, and these attributes were also related to DI symptoms.

Lazarus and Folkman's stress and coping (MSC) model suggests that individuals respond to stressors based on their perceived coping ability. The process involves three key components: the stressor, the individual's appraisal of the situation, and the coping strategies employed (Lazarus and Folkman, 1984). Medical illnesses like cancer is a cause of life-threatening stress, and most patients suffer from symptom burdens, such as digestive system dysfunction, organ dysfunction, fatigue, pain and an array of secondary stressors (Chang et al., 2022). The process of diagnosis, treatment, and rehabilitation of medical illness relies mainly on supportive family care and financial support. Managing concerns associated with medical illness requires individuals to use a variety of coping strategies.

Regarding the connection between demoralization and coping strategies, problem-solving was linked to reduced feelings of demoralization. Thus, by engaging in problem-solving, individuals can uphold their proactive, flexible, and tolerant attitudes towards stressors while also ensuring the recognition of internal resources like self-esteem, self-reliance, and mastery, safeguarding against demoralization. If patients lack social support and have coping difficulties, they may develop demoralization. (Bobevski et al. 2022)a thought a natural progression in the sequence of development of demoralization can be understood in that patients can recognize a sense of not coping well, which lowers their morale, leaves them feeling trapped, and causes distress. Loss of key roles lowered confidence, and inability to meet expectations contribute to this mental state.

Consequently, they may experience feelings of loneliness, helplessness, and hopelessness and lose self-esteem and the purpose of life, resulting in feelings that life is meaningless and contemplating suicide. The phenomenology of demoralization is consistent with our research findings; demoralization can be viewed as a product of a failed stress-coping process, where individuals perceive stressors as overwhelming, appraise their ability to cope as inadequate, and resort to ineffective coping strategies. The demoralized patient expresses dysphoria and disheartenment, helplessness, hopelessness, loss of meaning and purpose, and a sense of failure. These negative emotions may lead to poor quality of life, depression and anxiety, and even desire to die and suicidal ideation. Kissane et al. (Kissane et al., 2004a) thought that the development of demoralization can be traced from the initial feelings of dysphoria and disheartenment, leading to a sense of failure, helplessness, and loss of meaning and purpose. Disheartenment, dysphoria, and a sense of failure are the specific aspects of demoralization related to clinical symptoms that appear to have a stronger impact on the patients. These clinical symptoms are characterized by a state of discouragement, loss of confidence, emotional distress, and loss of sense of worth and efficacy, stemming from ineffective coping mechanisms or unmet wishes (Kissane, 2001). Bobevski et al. discovered that hopelessness and meaning were the most strongly linked to thoughts of death and suicide through the use of Exploratory Graph Analysis (EGA) (Bobevski et al., 2022b). Murri et al., using EGA, found that the community's “loss of purpose” included signs of hopelessness, loss of meaning, existential distress, and indicators of diminished self-esteem, another important aspect of demoralization (Murri et al., 2020). Li et al. also conducted a concept analysis of demoralization based on the Walker and Avant. They found five attributes: long-term exposure to a stressful event, a psychological state that lasts for a painful period, loss of control over life, feeling uncertain about the future, and loss of meaning and purpose (Li et al., 2015). However, in this study, it is a pity that the stressful event is identified as an attribute rather than an antecedent. Frankl thought that external stressors can lead to existential crises and feelings of demoralization (Frankl, 1985). Therefore, the stressful event can be seen as an antecedent.

In summary, demoralization results from the mechanism between stressors (medical illness, symptom burden, low social support) and the individual's coping mechanisms. When individuals perceive these stressors as overwhelming and feel coping difficulties to manage them, they may experience a cascade of negative emotions leading to a significant decline in mental health and quality of life, even suicidal ideation.

### 4.1 Implications for clinical practice

Numerous prominent researchers have suggested that establishing a diagnostic classification for demoralization may enhance awareness of the condition, dialogue between healthcare providers and individuals, and influence treatment decisions to improve quality of life (Robinson et al., 2016). Our concept analysis can delineate the boundaries of demoralization and provide insight into the true origins of distress and illness experiences that may not always be fully captured by diagnosing depression. In addition, recognizing demoralization earlier in clinical settings can improve risk evaluation and treatment effectiveness.

Antecedents offer a glimpse into the contextual factors that lead to the development of demoralization in health, including medical illness, symptom burden, low social support and coping difficulties. Demoralization can occur in any clinical situation, not just in life-threatening diseases like cancer, and is currently being examined in patients with Parkinson's disease, kidney transplant recipients, refugee and migrant populations, and the general population. These antecedents highlight the importance of context and situational factors. It encourages research into how different stressors impact individuals uniquely based on their circumstances, resources, and support systems. The six defining attributes and empirical referents of this concept could be used to assist healthcare providers in recognizing demoralization in patients in clinical practice. The examples provided in the present analysis could be used to identify and differentiate between borderline and related cases of demoralization in patients. Especially distinguishing between depression and demoralization. In DSM-5, loss of meaning and purpose or hopelessness is not included as key diagnostic criteria for a major depressive disorder (American Psychiatric Association and American Psychiatric Association, 2013). Major depression is firmly based on the feelings of losing interest and pleasure. However, being stuck in a situation that deprives one of anticipated meaning and purpose is a prime example of demoralization (Kissane, 2014). Different definitions of demoralization exist, and the prevalence of demoralization varies depending on the assessment tools utilized. Our analysis helps define and understand demoralization in depth within the health context. Therefore, the items should encompass all concept attributes to enhance the instruments’ accuracy. A crucial aspect of demoralization involves loss of meaning and purpose, and meaning therapy is designed to combat this issue. There were many studies on meaning-related psychotherapy, CALM therapy, and dignity therapy (DT), and they were created to assist cancer patients in finding meaning during and after their illness and to uphold a sense of meaning towards the end of life (Wang et al., 2023a). Logotherapy (logos = meaning) and individual meaning-centered therapy have a significant effect on increasing the level of demoralization (Wang et al., 2023b). It is also recognized that culture plays a large part in how illness and demoralization are experienced. A person from a collectivist society could feel ashamed upon hearing about poor health, while a person from an individualistic culture, like Australia, might feel demoralized in the same situation life (Robinson et al., 2016). What cultural and religious factors influence this? Such speculation requires further research. The consequences of demoralization include poor quality of life, depression and anxiety, and demoralization was found to contribute to an increased risk for suicidal ideation uniquely. Such findings may suggest that untreated demoralization can lead to extremely serious consequences for some patients. Therefore, healthcare professionals should take measures to assess the risk of demoralization and minimize its occurrence quickly. The DSM-5 should consider demoralization as the precursor of depression or put it as one category for healthcare providers to use to detect and provide treatment earlier.

### 4.2 Limitations

There are a few limitations to this study which must be considered. First, although this review employed a systematic and rigorous methodology, the meticulously formulated databases, search strategy, and inclusion criteria may still have resulted in the inadvertent exclusion of some pertinent studies not identified by the search. Second, most included studies were limited to cross-sectional studies, with few longitudinal studies or qualitative studies of demoralization. Future research will examine the long-term relationship between demoralization and associated factors.

Furthermore, qualitative research should be included to explore individual experiences with demoralization. This can provide a deeper insight into how individuals perceive their struggles and the cultural contexts surrounding them. Word cloud analysis was used, which can be problematic when conducting concept analyses because defining attributes are determined based on their repetition in the literature. Therefore, attributes that are mentioned less often but could be important tend to be ignored. We solely utilized the Walker and Avant concept analysis method to examine the concept of demoralization. The researcher's biases or perspectives may influence the method, potentially leading to varied interpretations of the same concept. Other techniques could offer more details to enhance the understanding of this concept. Scoping review without quality assessment of included studies may lead to some analyzed results not being backed by strong empirical evidence, impacting the usefulness of the findings.

## 5 Conclusion

Six decisive attributes were determined through the conceptual analysis of demoralization, including dysphoria, disheartenment, helplessness, hopelessness, loss of meaning and purpose, and a sense of failure. When patients experience demoralization, it is a key factor causing poor quality of life, depression, anxiety, suicidal ideation and desire to die. Demoralization is often underpinned by medical illness, symptom burden, low social support and coping difficulties. The concept analysis provides a theoretical framework for healthcare providers to understand demoralization in medical patients better and improve patient well-being.

## Funding

No external funding.

## CRediT authorship contribution statement

**Rongyu Hua:** Writing – original draft, Resources, Methodology, Formal analysis, Data curation, Conceptualization. **Patraporn Bhatarasakoon:** Writing – review & editing, Visualization, Validation, Supervision, Resources, Project administration, Methodology, Investigation, Data curation, Conceptualization.

## Declaration of competing interest

None.

## Figures and Tables

**Table:** 

Database	Search	Search strategy	Results
PubMed	#1	Demoralization [MeSH]	1119
	#2	demorali * [All Fields]	
	#3	#1 OR #2	
		Filters: Publication year (to 2024/02/15)	
Embase	#1	(demoralization or demoralization syndrome or demorali*) [All Fields]	1771
		Limits:Publication year (to 2024)	
PsycINFO	#1	(demoralization or demoralization syndrome or demorali*) [Abstract]	1083
	#2	(demoralization or demoralization syndrome or demorali*) [Tiltle]	
	#3	#1 OR #2	
		Limits:Publication year (to 2024)	
Web of Science	#1	(demoralization or demoralization syndrome or demorali*) [Title]	632
		Filters:Publication year (to 2024)	
Cochrane Library	#1	(demoralization or demoralization syndrome or demorali*) [Title Abstract Keyword]	105
		Limits:Publication year (to September 2024)	
